# Correlation analysis of recurrent factors in borderline ovarian tumors undergoing fertility preservation surgery

**DOI:** 10.3389/fonc.2025.1488247

**Published:** 2025-01-22

**Authors:** Yichi Xie, Dandan Wang, Ningning Zhang, Qing Yang

**Affiliations:** Department of Obstetrics and Gynecology, Shengjing Hospital of China Medical University, Shenyang, China

**Keywords:** borderline ovarian tumor, recurrence, fertility preservation surgery, high risk factor, surgical treatment

## Abstract

**Objective:**

To explore the relapse - related factors of fertility preservation surgery for borderline ovarian tumors.

**Methods:**

Patients of childbearing age who underwent fertility preservation surgery for borderline ovarian tumors in Sheng jing Hospital of China Medical University from April 20 1 8 to April 20 2 3 were selected. Clinical data were collected and their clinical characteristics were statistically analyzed. It is to explore the risk factors of postoperative recurrence.

**Results:**

A total of 30 8 patients were included in this study, of which 1 was lost to follow - up and 47 relapsed (4 7/3 0 7, 15. 3 1%). The results of multivariate analysis showed that the pathological features of micro papillary structure, intra operative as cites, bilateral tumors, and the increased ratio of neu tro phil to lymphocyte before surgery are independent risk factors for the recurrence of borderline ovarian tumors.

**Conclusion:**

The prognosis of women of childbearing age with borderline ovarian tumors undergoing conservation function surgery is good. However, patients with high - risk recurrence factors should be paid special attention and closely followed up after surgery.

## 1 Introduction

Borderline ovarian tumors (BOTs) have pathologic characteristics, biological behaviors, and prognosis between benign and malignant tumors (1).Six histologic subtypes of BOT are distinguished based on the epithelial cell type, similar to invasive carcinomas, comprising serous (50%) and mucinous (45%), and less common subtypes including endometrioid, clear cell, seromucinous,and borderline Brenner tumor (2). It accounts for about 15% of ovarian epithelial tumors (3). Surgical treatment is the main method of BOTs treatment. Most of the patients are young women of childbearing age and have fertility needs, so fertility preservation therapy is particularly important. The surgical approach of fertility preservation is open or laparoscopic exploration. The surgical methods were ovarian tumor excision, affected side adnexectomy, unilateral adnexectomy + contralateral ovary dissection/tumor excision, and staged operation with fertility preservation. The prognosis of patients with BOTs fertility preservation surgery is good, but some patients still have postoperative recurrence (4), and most tumor recurrence sites are located in ovaries. At present, there are few and controversial studies on relapse factors. Therefore, this study intends to analyze relevant clinical data to find out the risk factors for recurrence after BOTs, so as to provide guidance for clinical postoperative follow-up strategies and reproductive function preservation decisions, and enhance the understanding of BOTs.

## 2 Data and methods

### 2.1 Research object

A total of 895 patients with borderline ovarian tumors were selected from Shengjing Hospital Affiliated to China Medical University from April 2018 to April 2023, including 472 patients of childbearing age (18-45 years old) and 308 patients who underwent conservation function surgery. The 308 patients were selected as the study object, 1 case was lost to follow-up, and 307 patients were enrolled with complete clinical data and follow-up information.

Nearly half of the patients were asymptomatic, and all of them were treated with pelvic masses found by physical examination, and a few of them were treated with menstrual changes.

Inclusion criteria

(1) Women of childbearing age between 18 and 45
(2) Complete case data
(3) Patients undergoing fertility preservation surgery
(4) Regular postoperative follow-up
(5) Postoperative diagnosis of borderline ovarian tumor was made by 2 pathologists

Exclusion criteria

(1) No reproductive need
(2) No operation to preserve fertility function (complete hysterectomy, double appendices)
(3) Missing follow-up, incomplete case data
(4) Other malignant tumors

### 2.2 Collection methods

Through HIS medical record system of Shengjing Hospital Affiliated to China Medical University, clinicopathologic data such as age, maternity history, symptoms, imaging results, biochemical examination (tumor markers, etc.), operation method, intraoperative records, postoperative pathology (whether micropapillae or papillae were present, whether implants were present, whether ruptures were present) were collected. The prognosis and recurrence of patients were followed up.

### 2.3 Information standards

(1) According to laboratory diagnostic criteria CA125: > 4 7 U/ml is elevated, 0-47U/ml is normal CA199: > 30U/ml for elevated, 0-30U/ml for normal CA724: > 6.9U/ml is elevated, 0-6.9U/ml is normal HE4: > 140 is elevated, 0-140 is normal AFP: > 7 is elevated, 0-7 is normal CEA: > 5 is elevated, 0-5 is normal ROMA-Before: ≥11.4 indicates elevated and < 11.4 indicates normal ROMA-After: ≥29.9 is elevated, < 29.9 is normal
(2) Whether rupture, hemorrhage and necrosis, ascites were determined by the actual intraoperative records
(3) The limit of tumor size was 10cm (10cm was the median tumor size of 307 patients), and the limit of neutrophil to lymphocyte ratio was 2.48 (2.48 was the median ratio of the two in 307 patients).
(4) Surgical approaches were divided into laparoscopy and open surgery; The surgical methods were ovarian tumor excision, affected side adnexectomy, unilateral adnexectomy + contralateral ovary dissection/tumor excision, and staged operation with fertility preservation
(5) The follow-up period was from 10 months to 70 months, and the date was March 31, 2024. The criteria for recurrence were new pelvic mass or mass enlargement indicated by postoperative imaging with or without elevated tumor markers. The follow-up methods were outpatient, telephone and secondary hospital admission. The follow-up included prognosis, recurrence, whether there was follow-up treatment, and whether there was a second operation

### 2.4 Statistical methods

SPSS26.0 software was used for statistical analysis of the relevant data. The measurement data of the normal distribution were described by the standard deviation of the mean soil (x ± s), and the counting data were expressed by the example (%). The comparison between the two groups was conducted by Chi-square test. The significant index in the univariate analysis, that is, the factor with P < 0.05, was included in the multivariate binary logistic regression analysis.

## 3 Results

### 3.1 General information

As shown in **Table 1**, part of the general data included in the patients

**Table 1:** General information.

Influencing factor	Number	Percentage
**Pathological** serous	131	42.67%
mucinous	108	35.18%
seromucinous	45	14.66%
endometrioid	23	7.49%
**Range** focal borderline	127	41.37%
borderline	154	50.16%
focal malignant change	15	4.89%
Malignant	11	3.58%
**Hemorrhagic necrosis** yes	11	3.58%
no	296	96.42%
**Micropapillae** yes	13	4.23%
no	294	95.77%
**Papillae** yes	179	58.31%
no	128	41.69%
**ascites** yes	45	14.66%
no	262	85.34%
**rupture** yes	14	4.56%
no	293	95.44%
**grow** yes	10	3.26%
no	297	96.74%
**A history of pregnancy** yes	133	43.32%
no	174	56.68%
**Hemi** bilateral	44	14.33%
unilateral	263	85.67%
**Size** > 10cm	137	44.62%
≤10cm	170	55.38%
**Surgical approach** laparoscopy	140	45.60%
Open surgery	167	54.40%
**FIGO** stage I	292	95.11%
Stage II and above	15	4.89%
**Is there a blood flow signal?** yes	214	69.71%
no	93	30.29%
**Lymph node dissection** yes	35	11.40%
no	272	88.60%
**Omentectomy** yes	62	20.20%
no	245	79.80%
**An appendectomy** yes	67	21.82%
no	240	78.18%
**NLR** Large	153	49.84%
small	154	50.16%
**Surgical methods** Tumor nucleation	112	36.48%
Accessory cut	90	29.32%
Adnexal resection + contralateral ovarian tumor nucleation	57	18.57%
Staging operation to preserve fertility function	48	15.64%

Bold values indicates a more significant manifestation of influencing factor.

As shown in **Table 2**, according to the laboratory diagnostic criteria, among 307 patients, 300 patients underwent CA125 testing, and 140 patients had elevated levels; CA199 was detected in 278 patients, and the level of CA199 was increased in 83 patients. CA724 was detected in 280 patients, and 70 patients had elevated levels. HE4 detection was performed in 270 patients, and 11 cases were elevated. AFP was detected in 273 patients, and 15 cases were elevated. In 283 patients, CEA was detected, 20 cases were elevated. The pre - and post-Roma tests were performed in 256 patients, of which 79 were preROMA and 88 were postROMA.

**Table 2:** Tumor marker.

Tumor marker	Test number	Positive number	Negative number	Positive rate
CA125	300	140	160	46.67%
CA199	278	83	195	29.86%
CA724	280	70	210	25%
HE4	270	11	259	4.07%
AFP	273	15	258	5.49%
CEA	283	20	263	7.07%
premenopausal Rome index	256	79	177	30.86%
postmenopausal Rome index	256	88	168	34.38%

The surgical staging was carried out in accordance with the International Federation of Gynecology and Obstetrics (FIGO), intraoperative findings, and postoperative pathology. Most BOTs were identified as being at stage I (5).

There were 15 patients (4.89%, 15/307) with tumor stage II and above, and 292 patients at stage I, which was in line with literature reports.

### 3.2 Introduction to recurrence

Of 307 patients, 47 relapsed, with a recurrence rate of 15.31%. The recurrence rate reported in the literature is 10%-15% (6), which is roughly consistent with this range. The follow-up treatment of patients with recurrence is shown in **Table 3** below. Of the 32 patients who underwent supplementary operation, 31 cases were still borderline ovarian tumor and 1 case was malignant ovarian tumor. Up to now, 47 patients have no death, the survival rate is 100%, no lost to follow-up cases, follow-up is still continuing (**Table 3** for details).

**Table 3:** Relapse outcome.

Management of recurrent patients	Number
Conservative treatment	15
Tumor nucleation	7
Accessory cut	3
Adnexal resection + contralateral ovarian tumor nucleation	1
Staging operation to preserve fertility function	5
Total staging operation	16
mortality rate	0

### 3.3 Single factor analysis of clinical features of ovarian borderline tumors

The relevant data of 307 patients were statistically analyzed, and a total of 27 independent variables were included (**Table 4** for details). Through statistical single factor analysis, 19 indicators were significant, that is, statistically significant; Pathological type, lesion scope, tumor pathology with micropapillae, intraoperative ascites, intraoperative tumor rupture, tumor implantation, bilateral tumor, tumor size > 10cm, late FIGO stage, ultrasound indicating blood flow signal, surgical method, lymph node dissection, greater omentectomy, appendectomy, NLR elevated, CA125 elevated, CA724 elevated, The increase of HE4 and the increase of post-ROMA index were the risk factors for recurrence of BOTs (P < 0.05). The remaining 8 indicators, tumor hemorrhage and necrosis, maternal history, surgical approach, tumor papilla, CA199, AFP, CEA, and pre-ROMA index, were not statistically significant.

**Table 4:** Single factor regression analysis of influencing factors of BOTs recurrence.

Influencing factor	Recurrence	Non-recurrence	Chi-square value	significance
**Pathological** serous	29(22.14%)	102(77.86%)	14.982	0.009
mucinous	6(5.56%)	102(94.44%)		
seromucinous	7(15.56%)	38(84.44%)		
endometrioid	5(21.74%)	18(78.26%)		
**Range** focal borderline	9(7.09%)	118(92.91%)	17.836	0.008
borderline	34(22.08%)	120(77.92%)		
focal malignant change	4(26.67%)	11(73.33%)		
Malignant	0(0%)	15(100%)		
**Hemorrhagic necrosis** yes	3(27.27%)	8(72.73%)	1.073	0.272
no	44(14.86%)	252(85.14%)		
**Micropapillae** yes	11(84.62%)	2(15.38%)	33.049	0
no	36(12.24%)	258(87.76%)		
**Papillae** yes	33(18.44%)	146(81.56%)	3.342	0.075
no	14(10.94%)	114(89.06%)		
**ascites** yes	26(57.78%)	19(42.22%)	55.256	0
no	21(8.02%)	241(91.98%)		
**rupture** yes	8(57.14%)	6(42.86%)	13.837	0
no	39(9.90%)	264(90.10%)		
**grow** yes	7(70%)	3(30%)	15.857	0
no	42(14.14%)	255(85.86%)		
**A history of pregnancy** yes	26(19.55%)	107(80.45%)	3.222	0.074
no	21(12.07%)	153(87.93%)		
**Hemi** bilateral	23(52.72%)	21(47.73%)	41.253	0
unilateral	24(9.13%)	239(90.87%)		
**Size** > 10cm	13(9.42%)	125(90.58%)	6.972	0.011
≤10cm	34(20.12%)	135(79.88%)		
**Surgical approach** laparoscopy	17(12.14%)	123(87.86%)	2.02	0.161
Open surgery	30(17.96%)	137(82.04%)		
**FIGO** stage I	37(12.67%)	255(87.33%)	21.749	0
Stage II and above	10(66.67%)	5(33.33%)		
**Is there a blood flow signal?** Yes	40(18.69%)	174(81.31%)	6.967	0.016
no	7(7.53%)	86(92.47%)		
**Lymph node dissection** yes	14(40%)	21(60%)	14.668	0
no	33(12.13%)	239(87.87%)		
**Omentectomy** yes	19(30.65%)	43(69.35%)	12.266	0
no	28(11.43%)	217(88.57%)		
**An appendectomy** yes	17(25.37%)	50(74.63%)	6.069	0.011
no	30(12.5%)	210(87.5%)		
**NLR** Large	35(22.88%)	118(77.12%)	13.969	0
small	12(7.80%)	142(92.20%)		
**Surgical methods** Tumor nucleation	17(15.18%)	95(84.82%)	27.151	0
Accessory cut	9(10%)	81(90%)		
Adnexal resection + contralateral ovarian tumor nucleation	2(3.51%)	55(96.49%)		
Staging operation to preserve fertility function	19(39.58%)	29(60.42%)		
**CA125** rise	28(20%)	112(80%)	4.409	0.038
normal	18(11.25%)	142(88.75%)		
**CA199** rise	17(20.48%)	66(79.52%)	1.852	0.168
normal	27(13.85%)	168(86.15%)		
**CA724** rise	16(22.86%)	54(77.14%)	4.654	0.027
normal	25(11.90%)	185(88.10%)		
**HE4** rise	4(36.36%)	7(63.64%)	3.585	0.042
normal	34(13.13%)	225(86.87%)		
**AFP** rise	2(13.33%)	13(86.67%)	0.053	0.821
normal	40(15.50%)	218(84.50%)		
**CEA** rise	3(15%)	17(85%)	0.001	0.983
normal	39(14.83%)	224(85.17%)		
**premenopausal Rome index** rise	19(24.05%)	60(75.95%)	7.955	0.055
normal	18(10.17%)	159(89.83%)		
**postmenopausal Rome index** rise	19(21.59%)	69(78.41%)	5.286	0.021
normal	18(10.71%)	150(89.28%)		

Bold values indicates a more significant manifestation of influencing factor.

### 3.4 Multivariate analysis of clinical features of ovarian borderline tumors

(**Table 5** for details)The results of multi-factor analysis showed that the tumor had five indexes: micropapillae, intraoperative ascites, tumor side, NLR ratio > 2.48, and lesion range, and the significance was P<0.05. It can be concluded that the above 5 indexes are independent risk factors affecting the recurrence of BOTs.

**Table 5:** Multivariate regression analysis of influencing factors of BOTs recurrence.

Influencing factor	B	Standard error	Wald	significance	Exp(B)	95% confidence interval for Exp(B)	
						lower limit	Upper limit
**Pathological** serous	0.782	0.781	1.004	0.316	2.186	0.473	10.092
mucinous	1.226	0.875	1.963	0.161	3.409	0.613	18.948
seromucinous	0.773	0.938	0.68	0.41	2.167	0.345	13.618
endometrioid	0						
**Range** focal borderline	-19.977	1.151	301.418	0	2.11E-09	2.21E-10	2.01E-08
borderline	-20.357	1.055	372.571	0	1.44E-09	1.83E-10	1.14E-08
focal malignant change	-20.553	0			1.19E-09	1.19E-09	1.19E-09
Malignant	0						
**Micropapillae** yes	2.9	1.027	7.97	0.005	18.166	2.427	135.987
no	0						
**ascites** yes	2.737	0.584	21.979	0	15.438	4.917	48.472
no	0						
**rupture** yes	0.562	1.379	0.166	0.684	1.753	0.117	26.172
no	0						
**grow** yes	0.695	1.306	0.283	0.595	2.004	0.155	25.942
no	0						
**Hemi** bilateral	1.255	0.595	4.449	0.035	3.506	1.093	11.249
unilateral	0						
**Size** > 10cm	-0.884	0.602	2.151	0.142	0.413	0.127	1.346
≤10cm	0						
**FIGO** stage I	0.586	1.505	0.152	0.697	1.798	0.094	34.331
Stage II and above	0						
**Is there a blood flow signal?** Yes	0.966	0.629	2.354	0.125	2.626	0.765	9.017
no	0						
**Surgical methods** Tumor nucleation	2.028	1.338	2.297	0.13	7.602	0.552	104.777
Accessory cut	1.689	1.334	1.602	0.206	5.413	0.396	73.999
Adnexal resection + contralateral ovarian tumor nucleation	2.702	1.449	3.478	0.062	14.908	0.871	255.069
Staging operation to preserve fertility function	0						
**Lymph node dissection** yes	-0.567	1.004	0.318	0.573	0.567	0.079	4.06
no	0						
**Omentectomy** yes	-1.308	1.212	1.164	0.281	0.27	0.025	2.909
no	0						
**An appendectomy** yes	-0.519	1.003	0.267	0.605	0.595	0.083	4.251
no	0						
**NLR** Large	1.19	0.514	5.352	0.021	3.286	1.199	9.004
small	0						

Bold values indicates a more significant manifestation of influencing factor.

### 3.5 Serous borderline ovarian tumor recurrence univariate analysis

The relevant data of 131 SBOT patients were statistically analyzed, and a total of 18 independent variables were included (**Table 6** for details). Through statistical single factor analysis, all the 11 indicators were significant, that is, statistically significant; Tumor pathology with micropapillae, intraoperative ascites, intraoperative tumor rupture, tumor implantation, bilateral tumor, late FIGO stage, blood flow signal indicated by ultrasound, surgical method, lymph node dissection, appendectomy, and increased NLR were all risk factors for recurrence of BOTs (P < 0.05). However, the remaining 7 indicators, including lesion scope, hemorrhage and necrosis, tumor pathology, nipple, maternal history, tumor size, surgical approach, and omentectomy, were not statistically significant.

**Table 6:** Single factor regression analysis of influencing factors of SBOTs recurrence.

Influencing factor	Recurrence	Non-recurrence	Chi-square value	Significance
**Range** focal borderline	4	37	8.529	0.105
borderline	24	65		
focal malignant change	1	0		
Malignant	0	0		
**Hemorrhagic necrosis** yes	1	5	0.116	0.742
no	28	97		
**Micropapillae** yes	11	2	26.548	0
no	18	100		
**Papillae** yes	26	81	1.756	0.218
no	3	21		
**ascites** yes	14	6	26.152	0
no	15	96		
**rupture** yes	4	3	4.291	0.037
no	25	99		
**grow** yes	4	2	5.765	0.02
no	25	100		
**A history of pregnancy** yes	17	48	1.212	0.274
no	12	54		
**Hemi** bilateral	18	15	24.198	0.002
unilateral	11	87		
**Size** > 10cm	5	30	1.828	0.197
≤10cm	24	72		
**Surgical approach** laparoscopy	12	58	2.175	0.143
Open surgery	17	44		
**FIGO** stage I	23	98	7.349	0.007
Stage II and above	6	4		
**Is there a blood flow signal?** Yes	26	67	7.3	0.019
no	3	35		
**Lymph node dissection** yes	11	12	9.342	0.002
no	18	90		
**Omentectomy** yes	11	22	3.018	0.077
no	18	80		
**An appendectomy** yes	11	20	3.901	0.044
no	18	82		
**NLR** Large	21	48	6.027	0.019
small	8	54		
**Surgical methods** Tumor nucleation	12	43	20.439	0.035
Accessory cut	4	24		
Adnexal resection + contralateral ovarian tumor nucleation	0	21		
Staging operation to preserve fertility function	13	14		

Bold values indicates a more significant manifestation of influencing factor.

### 3.6 Multivariate analysis of clinical features of serous borderline ovarian tumors

(**Table 7** for details)The results of multi-factor analysis showed that the tumor had micropapillae, intraoperative ascites, ultrasound blood flow signal, and NLR ratio > 2.48 (P<0.05). It can be concluded that the above 4 indexes are independent risk factors affecting the recurrence of SBOTs.

**Table 7:** Multivariate regression analysis of influencing factors of SBOTs recurrence.

Influencing factor	B	Standard error	Wald	significance	Exp(B)	95% confidence interval for Exp(B)	
						lower limit	Upper limit
**Micropapillae** yes	3.877	1.237	9.823	0.002	48.285	4.274	545.49
no	0						
**ascites** yes	3.078	0.868	12.576	0	21.706	3.962	118.935
no	0						
**rupture** yes	-0.577	1.804	0.102	0.749	0.561	0.016	19.266
no	0						
**grow** yes	-2.979	1.68	3.142	0.076	0.051	0.002	1.37
no	0						
**Hemi** bilateral	1.641	0.866	3.59	0.058	5.16	0.945	28.173
unilateral	0						
**FIGO** Stage II and above	1.122	1.733	0.42	0.517	3.072	0.103	91.749
Stage I	0						
**Is there a blood flow signal?** Yes	3.581	1.517	5.573	0.018	35.914	1.837	702.189
no	0						
**Surgical methods** Tumor nucleation	3.838	2.586	2.202	0.138	46.442	0.292	7386.022
Accessory cut	3.422	2.648	1.67	0.196	30.635	0.171	5497.42
Adnexal resection + contralateral ovarian tumor nucleation	24.801	0			58986206330	58986206330	58986206330
Staging operation to preserve fertility function	0						
**Omentectomy** yes	-1.126	1.641	0.471	0.493	0.324	0.013	8.084
no	0						
**An appendectomy** yes	-2.675	1.991	1.806	0.179	0.069	0.001	3.41
no	0						
**NLR** Large	1.791	0.837	4.586	0.032	5.997	1.164	30.904
small	0						

Bold values indicates a more significant manifestation of influencing factor.

### 3.7 Single factor analysis of recurrence of Mucinous borderline ovarian tumors

The relevant data of 108 MBOTs patients were statistically analyzed, and a total of 17 independent variables were included (**Table 8** for details). Through statistical single factor analysis, all the three indexes were significant, that is, statistically significant; Ascites, tumor implantation and late FIGO stage were all risk factors for recurrence of MBOTs (P < 0.05). However, the other 14 indicators, including lesion scope, hemorrhage and necrosis, tumor pathology including papilla, maternal history, tumor rupture, tumor lateralization, tumor size, surgical approach, surgical method, lymphadenectomy, greater omentectomy, appendectomy, NLR elevation, and ultrasound indicating blood flow signal, were not statistically significant.

**Table 8:** Single factor regression analysis of MBOTs relapse influencing factors.

Influencing factor	Recurrence	Non-recurrence	Chi-square value	Significance
**Range** focal borderline	3	62	3.373	0.437
borderline	1	22		
focal malignant change	2	9		
Malignant	0	9		
**Hemorrhagic necrosis** yes	0	2	0.231	0.9
no	6	100		
**Papillae** yes	0	27	3.568	0.99
no	6	75		
**ascites** yes	5	9	17.02	0.001
no	1	93		
**rupture** yes	1	2	2.322	0.078
no	5	100		
**grow** yes	1	1	3.272	0.043
no	5	101		
**A history of pregnancy** yes	3	32	0.843	0.354
no	3	70		
**Hemi** bilateral	0	2	0.31	0.9
unilateral	6	100		
**Size** > 10cm	3	75	1.408	0.228
≤10cm	3	27		
**Surgical approach** laparoscopy	1	37	1.072	0.348
Open surgery	5	65		
**FIGO** Stage II and above	5	101	3.272	0.043
stage I	1	1		
**Is there a blood flow signal?** Yes	5	67	0.889	0.389
no	1	35		
**Lymph node dissection** yes	1	5	7.644	0.621
no	5	97		
**Omentectomy** yes	2	11	1.032	0.254
no	4	91		
**An appendectomy** yes	2	20	2.013	0.124
no	4	82		
**NLR** Large	3	47	0.585	0.426
small	3	55		
**Surgical methods** Tumor nucleation	3	33	0.035	0.852
Accessory cut	0	41		
Adnexal resection + contralateral ovarian tumor nucleation	1	20		
Staging operation to preserve fertility function	2	8		

Bold values indicates a more significant manifestation of influencing factor.

### 3.8 Multivariate analysis of clinical features of mucinous borderline ovarian tumors

(**Table 9** for details)The results of multi-factor analysis showed that there was significant ascites (P<0.05). It can be concluded that ascites is an independent risk factor for the recurrence of MBOTs.

**Table 9:** Multi-factor regression analysis of MBOTs relapse influencing factors.

Influencing factor	B	Standard error	Wald	significance	Exp(B)	95% confidence interval for Exp(B)	
						lower limit	Upper limit
**ascites** yes	3.746	1.214	9.52	0.002	42.335	3.921	457.137
no							
**FIGO** Stage II and above	0.921	1.561	0.348	0.555	2.511	0.118	53.49
Stage I							
**grow** yes	2.793	2.174	1.651	0.199	16.336	0.231	1157.752
no							

Bold values indicates a more significant manifestation of influencing factor.

## 4 Discussion

The above is all about the correlation analysis of surgical recurrence factors in patients with ovarian borderline tumors who preserve fertility function in this study. In recent years, the incidence of BOTs has shown a younger tren d, and the incidence has increased (7). Compared with benign tumors and malignant tumors, the correct diagnosis of ovarian borderline tumors is more difficult, but both have a good prognosis. According to literature reports, 75% of women are still in FIGO I stage when diagnosed with ovarian borderline tumors (8). Therefore, for women of childb earing age and with reproductive needs, nursing function surgery is still a better choice. Although patients with ovar ian borderline tumors undergoing fertility preservation surgery have a certain probability of recurrence, due to their good tumor behavior,secondary supplementary surgery can still achieve a better survival outcome (9). In this study, most of the major sites of ovarian borderline tumor recurrence were in the ovary, which was confirmed by other for eign studies (10, 11). The results of this study showed the pathological type, lesion scope, tumor pathology with microp apillary component, intraoperative ascites, intraoperative rupture of tumor, tumor implantation, bilateral tumor, tum or size > 10cm, late FIGO stage, ultrasonography indicating blood flow signal, surgical method, lymph node dissection, greater omentectomy, appendectomy, elevated NLR, and CA125 Increased levels of CA724, HE4 and post-ROMA index were all risk factors for recurrence of BOTs (P < 0.05). The results of multi-factor analysis showed that the tumor had micropapillary component, ascites was visible during operation, bilateral tumor, NLR ratio > 2.48, and lesion range, and the significance was P<0.05. It can be concluded that the above 5 indexes are independent risk factors affecting the recurrence of BOTs.

### 4.1 Pathological types and risk of recurrence after BOTs surgery

Ovarian borderline tumors include mucinous, serous, endometrioid, clear cell, Brenner tumor and other histological types (2). The 5-year data included in this study did not include clear cell and histological type of Brenner tumor, and the proportion was serous > mucous > seromucinous > endometrioid, which was generally in line with the classification criteria of female genital organ tumors in the 4th edition of WHO in 2014. The main histological types of ovarian borderline tumors are serous and mucous, with serous accounting for about 55% and mucous accounting for about 42% (12). Ovarian mucous borderline tumors include two subtypes, gastrointestinal type and cervical mucous type. Serous borderline ovarian tumors include transtypical and micropapillary types.

Micropapillary subtypes are special types of ovarian serous borderline tumors, most of which show bilateral involvement, ovarian surface involvement, invasive implantation, etc., and the prognosis is worse than that of common ovarian serous borderline tumors (13). In this study, univariate and multivariate logistic regression analysis showed that micropapillae were independent risk factors for the recurrence of borderline ovarian tumors. A retrospective study of 200 patients with long-term follow-up on the micropapillary type of ovarian serous borderline tumors also had the same view (14). In this study, of the 13 patients with micropapillary subtypes, 11 relapsed, and the relapse of these 11 patients was still borderline. It has been reported that ovarian serous borderline neoplasms have similar molecular and genetic changes to low-grade serous carcinoma (15–18). In some cases, there may be persistent tumor progression from cystadenoma and ovarian borderline tumors to low-grade cancers with shared clonal and MAPK pathway mutations, suggesting that the micropapillary type of ovarian serous borderline tumors is a pattern of progressive progression from ovarian serous borderline tumors to invasive ovarian cancers (19). Therefore, women of childbearing age who have borderline ovarian tumors with micropapillae subtypes and have fertility needs should complete fertility as soon as possible after fertility preservation surgery, and close follow-up should be conducted at this stage. Then, it was considered to return to the hospital for comprehensive staging surgery to obtain a good prognosis and survival outcome.

### 4.2 Surgical methods and risk of recurrence of BOTs

There are four surgical methods to preserve fertility function for borderline ovarian tumors: ovarian tumor excision, affected side adnexectomy, unilateral adnexectomy + contralateral ovary dissection/tumor excision, and staged operation to preserve fertility function. COX regression results of a systematic retrospective study in Germany showed that fertility preservation surgery was an independent risk factor for ovarian borderline tumor recurrence, but the study concluded that fertility preservation surgery could still be considered a safe choice for patients with early FIGO BOTs (20). Among the 307 patients included in this study, there were no deaths during the follow-up, and the survival rate was 100%. Therefore, although there was a certain recurrence rate, fertility preservation surgery was still the preferred treatment for young patients. It should be noted that comprehensive staging surgery may cause intestinal damage and urinary system damage (21), and the incidence of postoperative pain, pelvic adhesion, and infertility will increase, seriously affecting patients’ quality of life. Although the recurrence rate has been improved, the survival rate and quality of life of patients have not been affected, so fertility preservation surgery is still a feasible treatment. For patients with no need for fertility, NCCN guidelines in the United States point out that standard ovarian cancer surgery, staging surgery or tumor cell reduction should be performed (13), and Chinese guidelines also point out that comprehensive staging surgery is recommended for patients with implantation and no need for fertility.

The surgical approach in this study was not statistically significant by univariate analysis (P > 0.05). Many studies have shown that laparoscopic surgery has the advantages of less blood loss, short hospital stay, and early postoperative recovery (22–25). Expert consensus has suggested that laparoscopic surgery is the first choice for early BOTs patients (13), but laparoscopic surgery may lead to intraoperative rupture of tumors, such as trocar puncture, TROCA puncture, and incisions for tumor metastasis, thus increasing the incidence of postoperative recurrence (26, 27). The reason why this index is not significant in this study may be related to the relatively insufficient sample size of the single-center study. A study by Andrea Maneo (27) showed that laparoscopic surgery was selected when the tumor diameter was less than 5cm. In this study, univariate analysis and multivariate logistic regression analysis were performed on tumor size, and tumor size > 10cm was a risk factor for the recurrence of ovarian borderline tumors. This may be related to the fact that the tumor is larger and thus more likely to rupture, spread, and then relapse.

### 4.3 Intraoperative situation, tumor markers and risk of recurrence of BOTs

Neutrophil to lymphocyte ratio, NLR, is an indicator of inflammatory status and a marker of postoperative complications and prognosis. At present, NLR has been proven to be a strong prognostic factor for esophageal cancer, colon cancer, lung cancer, uterine sarcoma and other tumors (28–31). However, there is no uniform standard for truncation values with NLR. There has been a lot of research on NLR, but the consensus is not unanimous. In this study, 2.48, the median NLR of included patients, was used as the limit for univariate analysis, and the results showed significant results. Multivariate logistic regression analysis showed that NLR elevation was and independent risk factor for ovarian borderline tumor recurrence. For patients diagnosed with borderline ovarian tumor after surgery, preoperative NLR is higher, which should be paid more attention.

As for tumor markers, this study included CA125, CA199, CA724, HE4, AFP, CEA, ROMA premenopausal and postmenopausal indexes. CA125 is a common monitoring marker for ovarian cancer, and an elevated level of CA125 may also appear in some benign diseases, such as peritonitis, cirrhosis, endometriosis, etc. (32, 33). However, the specificity and sensitivity in the preliminary diagnosis and differentiation of benign and malignant ovarian masses are not ideal (34). The concentration of CA199 in the serum of patients with digestive tract tumors is significantly increased, and the diagnostic value is clear (35). In recent years, more and more studies have pointed out that the proportion of CA199 in mucinous non-benign tumors is increasing, and the proportion in this study is 24.49% (24/98), which seems not obvious, which may be related to the fact that this study is a single-center retrospective study. And the sample size is small. CA724 is a mucin-like high molecular weight glycoprotein, which is often used in the detection of malignant tumors, and is a non-specific tumor marker. It has high expression level in the serum of patients with gastric cancer, colorectal cancer and other tumors (36). Some studies have proved that CA724 expression is highly specific in the diagnosis of ovarian cancer, especially in the diagnosis of mucinous ovarian cancer (37). However, this study can only prove that the increase of CA724 is a risk factor for the recurrence of ovarian borderline tumors, but it is not an independent risk factor, and the specificity is not obvious. HE4 is a tumor marker found in recent years that can be used in the diagnosis of ovarian cancer. The level of HE4 is overexpressed in ovarian tumors. Its specificity is 94% (38). But it is often used to assist in the diagnosis of ovarian cancer.The increase of CEA in colon cancer and ovarian cancer (39, 40) was not significantly associated with borderline ovarian tumors, which was also confirmed by the statistical analysis in this study (P > 0.05), which was not significant. AFP, alpha-fetoprotein, is closely related to liver malignant tumors (41) and germ cell tumors to a certain extent, which is of certain value in the diagnosis of ovarian malignant tumors. However, the prediction and verification of the recurrence of ovarian borderline tumors are indeed limited, and further studies need more data from larger samples for evidence. In many studies, it has been confirmed that the pre - and post-ROMA index has a certain correlation with the diagnosis and recurrence of ovarian cancer, and the sensitivity and specificity are high.

Ascites provides an environment for tumor, promotes the proliferation of tumor cells and inhibits apoptosis (42). Ovarian tumor cells shed in ascites, survive and proliferate in the ascites microenvironment, and undergo morphological changes to enhance their proliferation and migration ability. When tumor cells leave ascites, it can be observed that their proliferation ability is significantly weakened. In ovarian cancer, ascites occurs in a considerable proportion of patients, and the prognosis is poor, and recurrence is often accompanied by ascites. In the multivariate logistic regression analysis, ascites was an independent risk factor for the recurrence of borderline ovarian tumors. Therefore, in patients with ovarian borderline tumors combined with ascites, close monitoring should be performed after conservation function surgery, and comprehensive staging surgery should be supplemented if necessary.

## 5 Conclusion

Taken together, borderline ovarian tumors are usually diagnosed at a younger age, which underscores the importance of considering fertility preservation approaches, and the prognosis is generally good. However, some risk factors may increase the likelihood of recurrence or malignant transformation (20). The postoperative pathological features of micropapillary structure, intraoperative ascites, bilateral tumors, and the increased ratio of neutrophils to lymphocytes before surgery are independent risk factors for the recurrence of ovarian borderline tumors, which should be paid special attention to. Therefore, the risk factors must be carefully considered and analyzed in order to adopt an appropriate surgical method to preserve fertility. Therefore, in combination with the clinic, the fertility needs of patients should also be considered, and the long-term prognosis and complications should be accounted for before surgery. Patients with related risk factors should indeed be carefully evaluated before surgery, and should also be paid attention to and closely followed up after surgery, encourage active completion of fertility, and supplement surgery if necessary.

## Data Availability

The original contributions presented in the study are included in the article/supplementary material. Further inquiries can be directed to the corresponding author.
